# Prevalence of hepatitis B virus marker positivity and evolution of hepatitis B virus profile, during chemotherapy, in patients with solid tumours

**DOI:** 10.1038/sj.bjc.6690652

**Published:** 1999-09

**Authors:** C G Alexopoulos, M Vaslamatzis, G Hatzidimitriou

**Affiliations:** Department of Medical Oncology and; Blood Transfusion Unit, Evangelismos Hospital, 45 Ipsilantou Str, Athens, 106 76, Greece

**Keywords:** HBsAg carriers, HBV profile, cancer chemotherapy

## Abstract

To prospectively evaluate the prevalence of hepatitis B virus (HBV) positivity and study the evolution of HBV profile during cancer chemotherapy, serum HBV markers and liver biochemistry were determined in 1008 of 1402 (72%) cancer patients admitted in our Unit and in all 920 (91%) who received chemotherapy. We found that 54 (5.3%) were HBsAg carriers while 443 (44%) had at least one HBV marker positive. Of the latter, 405 (91%) were HBcAb+ve, 321 (72%) HBsAb+ve and 212 (48%) HBeAb+ve. No patient was HBeAg+ve. Among 920 chemotherapy receivers, 374 (41%) were HBcAb+ve, 280 (30%) HBsAb+ve and 178 (19%) HBeAb+ve. Fifty (5.4%) were HBsAg carriers (versus 0.6% in Greek blood donors). All 50 were systematically screened for HBsAg and HBsAb status throughout chemotherapy, during follow-up or until their death, and liver biochemistry was performed before each chemotherapy course. Stable antigenaemia was observed in 43/50 (86%) while 7/50 (14%) developed clinical and/or biochemical hepatitis. Six of these seven developed serum anti-HBs antibodies with an associated decrease of serum HBsAg titres. We conclude that reactivation of HBV infection during chemotherapy is not rare (14%), while disappearance of HBs antigenaemia is neither a frequent nor usually a permanent phenomenon. © 1999 Cancer Research Campaign


British Journal of Cancer (1999) **81**(1), 69–74
© 1999 Cancer Research Campaign
Article no. bjoc.1999.0652

### Prevalence of hepatitis B virus marker positivity and evolution of hepatitis B virus profile, during chemotherapy, in patients with solid tumours

**CG Alexopoulos[1], M Vaslamatzis[1] and G Hatzidimitriou[2]**

[1]Department of Medical Oncology and [2]Blood Transfusion Unit, Evangelismos Hospital, 45 Ipsilantou Str, Athens 106 76, Greece

**Summary** To prospectively evaluate the prevalence of hepatitis B virus (HBV) positivity and study the evolution of HBV profile during cancer chemotherapy, serum HBV markers and liver biochemistry were determined in 1008 of 1402 (72%) cancer patients admitted in our Unit and in all 920 (91%) who received chemotherapy. We found that 54 (5.3%) were HBsAg carriers while 443 (44%) had at least one HBV marker positive. Of the latter, 405 (91%) were HBcAb+ve, 321 (72%) HBsAb+ve and 212 (48%) HBeAb+ve. No patient was HBeAg+ve. Among 920 chemotherapy receivers, 374 (41%) were HBcAb+ve, 280 (30%) HBsAb+ve and 178 (19%) HBeAb+ve. Fifty (5.4%) were HBsAg carriers (versus 0.6% in Greek blood donors). All 50 were systematically screened for HBsAg and HBsAb status throughout chemotherapy, during follow-up or until their death, and liver biochemistry was performed before each chemotherapy course. Stable antigenaemia was observed in 43/50 (86%) while 7/50 (14%) developed clinical and/or biochemical hepatitis. Six of these seven developed serum anti-HBs antibodies with an associated decrease of serum HBsAg titres. We conclude that reactivation of HBV infection during chemotherapy is not rare (14%), while disappearance of HBs antigenaemia is neither a frequent nor usually a permanent phenomenon.

**Keywords:** HBsAg carriers; HBV profile; cancer chemotherapy

Reactivation of hepatitis B virus (HBV) infection in patients with haematologic malignancies receiving cytotoxic or immunosuppressive therapy is well documented (Wands et al, 1975; Lau et al, 1989; Pinto et al, 1990; Ohtsu et al, 1991). It is of interest that in some of those HBsAg carriers who developed hepatitis during cytotoxic treatment, seroconversion to an HBsAg-negative state was observed (Scullard et al, 1981; Hoofnagle et al, 1982; Alexopoulos et al, 1992).

Information of this kind, in HBsAg-positive patients with solid tumours, treated with cytotoxic chemotherapy, is very limited and consisted mainly of case reports (Gallbraith et al, 1975; Ohtsu et al, 1991; Pinto et al, 1990; Alexopoulos et al, 1992). We therefore decided to undertake a systematic prospective study of the HBV profile in such patients, with a twofold aim:

1. to evaluate the prevalence of HBV marker positivity in patients with solid tumours
2. to study the evolution of HBV profile and the behaviour of HBsAg carrier state during anti-neoplastic chemotherapy.

Our findings have been previously presented, in abstract form, in the 33nd Annual Meeting of the American Society of Clinical Oncology (Alexopoulos et al, 1997).

### PATIENTS AND METHODS

### Prevalence of HBV markers positivity

#### *Patient population*

Between 1986 and 1995, 1402 patients with various solid tumours were admitted to the Department of Medical Oncology at Evangelismos Hospital. In 1008 of them (72%) serum markers for HBV infection were determined before any anticancer chemotherapy was given (Group A).

A total of 920 patients (91%) of Group A received antineoplastic chemotherapy (Group B) and they served as the main focus in studying the prevalence of marker positivity. The decision for not giving chemotherapy in the remaining 88 patients was based solely on the absence of reasonably effective chemotherapy for their tumour, their performance status and the presence of specific medical contra-indications. No patient was excluded on the basis of the liver status.

#### *Methods*

Serum markers for HBV infection, including HBsAg, HBeAg, HBsAb, HBcAb, and HBeAb, were determined using specific third-generation enzyme-linked immunosorbent assay (ELISA; Abbott), at the time of initial presentation in all patients (Group A) and it was repeated before chemotherapy in the 920 patients of Group B. Positive results were repeated for confirmation. Liver function tests including SGOT, SGPT, $\gamma$GT, LDH, alkaline phosphatase, bilirubin, phrothrombin time and serum proteins were also performed at the same time as HBV profile, before the administration of any treatment.

### Evaluation of HBV profile and behaviour of HBsAg carrier state during anti-neoplastic chemotherapy

#### *Patient population*

Among the 920 patients who received chemotherapy, 538 (59%) patients had HBV markers determined before and after the completion of their chemotherapy (Group C), while in 391 (43%) patients HBV markers were serially determined before chemotherapy, just after chemotherapy and during their follow-up

*Received 22 July 1998*
*Revised 15 January 1999*
*Accepted 12 April 1999*

*Correspondence to:* CG Alexopoulos

(Group D). These two groups of patients served as the focus in studying the evolution of HBV profile during antineoplastic chemotherapy.

There were 50 HBsAg carriers among the 920 patients of Group B and all of them were systematically screened for HBsAg and HBsAb status throughout chemotherapy, during their follow-up or until their death. Finally, in 30 of the 50 (60%) HBsAg carriers, we had the opportunity to monitor their HBV profile before, in the middle, just after the completion of chemotherapy and repeatedly during their follow-up. Both these groups served as the focus in studying the behaviour of HBsAg carrier status during cancer chemotherapy.

### Methods

Clinical and laboratory monitoring was as follows:

- History was taken and clinical examination was performed before each course of chemotherapy, during treatment and every 2 months during follow-up.
- Serum HBV marker determined before, in the middle, just after the completion of chemotherapy and during the follow-up every 2 months, for 1 year.
- Liver function tests including SGOT, SGPT, γGT, LDH, alkaline phosphatase, bilirubin, prothrombin time and serum proteins were performed before each course of chemotherapy during treatment, and every 2 months during follow-up, for 1 year.

### Statistics

Chi-square test was used for comparison of findings.

**Table 1** Distribution of the various types of cancer and prevalence of HBsAg carrier state in the 1008 patients, screened before any anticancer treatment was given (Group A)

| Type of cancer | Men (%) | Women (%) | Total | HbsAg +ve (%) |
|---|---|---|---|---|
| Carcinoma of the lung | 196 | 35 | 231 | 15 (6.5) |
| Carcinoma of the breast | 1 | 260 | 261 | 8 (3) |
| Carcinoma of the GI tract[a] | 103 | 67 | 170 | 7 (4.1) |
| HD and NHL | 55 | 58 | 113 | 14 (12.4) |
| Urothelial cancer | 59 | 27 | 86 | 4 (4.7) |
| Head and neck cancer | 10 | 6 | 16 | 2 (12.5) |
| Cancer of the ovary | – | 85 | 85 | 4 (4.7) |
| Other cancers | 35 | 11 | 46 | 0 |
| Total | 460 (45.6) | 548 (54.4) | 1008 | 54 (5.36) |

[a]Excluding hepatocellular carcinoma.

**Table 2** Distribution of the various types of cancer and prevalence of HbsAg carrier state in the 920 patients who received anticancer chemotherapy (Group B)

| Type of cancer | Men (%) | Women (%) | Total | HbsAg +ve (%) |
|---|---|---|---|---|
| Carcinoma of the lung | 176 | 25 | 201 | 14 (6.9) |
| Carcinoma of the breast | 1 | 225 | 226 | 6 (2.65) |
| Carcinoma of the GI tract[a] | 90 | 59 | 149 | 6 (4) |
| HD and NHL | 55 | 58 | 113 | 14 (12.4) |
| Urothelial cancer | 59 | 27 | 86 | 4 (4.7) |
| Head and neck cancer | 10 | 4 | 14 | 2 (14.3) |
| Cancer of the ovary | – | 85 | 85 | 4 (4.7) |
| Other cancers | 35 | 11 | 46 | 0 |
| Total | 426 (46.4) | 494 (53.6) | 920 | 50 (5.4) |

[a]Excluding hepatocellular carcinoma.

### RESULTS

### Patients characteristics

Among the 1008 patients tested before any treatment was given (Group A), 460 (45.6%) were men and 548 (54.4%) women with a median age of 59 years (17–78) compared with 426 (46.3%) men and 494 (53.6%) women with a median age of 58 years (19–78), in the 920 patients who subsequently received anticancer chemotherapy (Group B). The distribution of the various malignant solid tumours in the two groups (A and B) together with the prevalence of HbsAg carriers in each type of malignant tumour are shown in Tables 1 and 2.

### Prevalence of HBV marker positivity

The prevalence of HBV markers in each of the four groups (A, B, C and D) is shown in Table 3. In Group A, a total of 443 patients (44%) had at least one marker positive, indicating previous exposure to HBV infection. Fifty-four patients (5.3%) were found to be HBsAg carriers, and 321 patients (32%) were HBsAb-positive. No patient was found to be HBeAg-positive, while 212 (21%) were HBeAb-positive. Finally, 405 patients (40%) had HBc antibodies in their sera, representing 91% of those screened positive for at least one marker. None of the 443 patients with at least one HBV marker positive was demonstrating clinical or biochemical evidence of hepatitis (data not shown).

The pre-chemotherapy prevalence of HBV marker positivity in Group B was not statistically different from that of Group A (Table 3). More specifically, the prevalence of HBsAg carriers was 5.4%, and a total of 396 patients (43%) had at least one HBV marker positive. Of them, 94% were HBcAb-positive. Similarly, pre-chemotherapy findings concerning the prevalence of HBsAg carrier state, HBcAb positivity and the presence of at least one HBV marker positive were not significantly different in Groups C and D (Table 3). On the contrary, comparison between the four groups demonstrated a significantly ($P < 0.001$) lower prevalence of serum anti-HBe antibodies in Groups C and D and of anti-HBs antibodies in Group D (Table 3).

### Evolution of HBV profile and HBsAg carrier behaviour during anti-neoplastic chemotherapy

Table 4 summarizes the findings concerning the changes of HBV profile just after chemotherapy in the 538 patients of Group C. For each HBV marker, the overall seroconversion rate was calculated by dividing the absolute number of changes observed in the corresponding marker with the total number of patients screened (Table 4). Among 37 HBsAg carriers, one had lost HBs antigenaemia after six courses of chemotherapy while no change was observed in the status of 501 HBsAg-negative patients for an overall seroconversion rate of 0.2% for HBsAg marker. On the other hand, among 151 HBsAb-positive patients, five lost their HBs antibodies after six courses of chemotherapy, while 15 of the remaining 387 HBsAb-negative patients developed HBs antibodies in their sera. This represents a 3.7% seroconversion rate for HBsAb marker. The corresponding overall seroconversion rates,

 © 1999 Cancer Research Campaign

**Table 3** Prevalence of HBV markers in 1008 patients tested at presentation (Group A), 920 patients who received chemotherapy (Group B), 538 patients tested before and after chemotherapy (Group C) and 391 patients with serial marker determination before and after chemotherapy and during follow-up (Group D). Results of comparisons between the four groups, for each HBV marker, are shown below with asterisks

| Patient population | Patient no. | HBs Ag+ (%) | Hbe Ag+ (%) | HBs Ab+ (%) | Hbe Ab+ (%) | HBc Ab+ (%) | Any +ve (%) |
|---|---|---|---|---|---|---|---|
| Group A | 1008 | 54 (5.3) | 0 | 321 (32) | 212 (21) | 405 (40) | 443 (44) |
| Group B | 920 | 50 (5.4)[a] | 0 | 280 (30)[a] | 178 (19)[a] | 374 (41)[a] | 396 (43)[a] |
| Group C | 538 | 37 (6.9)[a] | 0 | 151 (28)[a] | 80 (15)[b] | 195 (36)[a] | 215 (40)[a] |
| Group D | 391 | 30 (7.7)[a] | 0 | 86 (22)[b] | 50 (13)[b] | 150 (40)[a] | 157 (40)[a] |

[a]Statistically non-significant; [b]statistically significant.

**Table 4** Changes in HBV profile in 538 patients screened before and just after chemotherapy (Group C)

| HBV marker | Before chemo | After chemo | Absolute number of changes (%) | Overall seroconversion rate[a] |
|---|---|---|---|---|
| HBsAg +ve | 37 | 36 | 1 | 1 (0.2) |
| HBsAg –ve | 501 | 501 | 0 | |
| HBsAb +ve | 151 | 146 | 5 | 20 (3.7) |
| HBsAb –ve | 387 | 372 | 15 | |
| HBeAg +ve | 0 | 0 | 0 | None |
| HBeAg –ve | 538 | 538 | 0 | |
| HBeAb +ve | 80 | 77 | 3 | 13 (2.4) |
| HBeAb –ve | 458 | 448 | 10 | |
| HBcAb +ve | 195 | 195 | 0 | 25 (4.6) |
| HBcAb –ve | 343 | 318 | 25 | |

[a]Absolute number of changes divided by the total number of patients (538) screened.

**Table 5** Evolution of HBV profile in 391 patients serially tested before, just after chemotherapy, and during their follow-up (Group D)

| HBV marker | Before chemotherapy | After chemotherapy | During follow-up | Overall change % |
|---|---|---|---|---|
| HBsAg +ve | 30 | 30 | 28 | 6.6 |
| HBsAb +ve | 86 | 83 | 82 | 4.7 |
| HBeAg +ve | 0 | 0 | 0 | None |
| HBeAb +ve | 50 | 53 | 58 | 16 |
| HBcAb +ve | 150 | 159 | 170 | 13.3 |

for HBeAb and HBcAb markers, were 2.4% and 4.6% respectively. No change whatsoever was observed in relation to HBeAg status.

The evolution of HBV profile, in 391 patients in whom HBV markers were serially determined before, just after chemotherapy and during follow-up (Group D), is presented in Table 5. Two of 30 HBsAg-positive patients became HBsAg-negative during follow-up, for an overall change of 6.6%. Among 86 HBsAb-positive patients, three lost their HBs antibodies after six courses of chemotherapy and another one during follow-up, for an overall change of 4.7%. Major changes were also observed in HBeAb and HBcAb status. Thus, while before chemotherapy 50 patients screened positive for HBeAb, the corresponding numbers were 53 after six courses and 58 during follow-up for an overall change of 16%. Likewise, nine patients developed HBc antibodies after six courses of chemotherapy and another 11 during follow-up, for an overall change of 13.3% (Table 5). Finally, serum HBV antigenaemia remained unchanged in 43 of 50 (86%) HBsAg carriers, in all measurements performed, and none of these patients developed clinical signs of hepatitis or liver function abnormalities. Nevertheless, in seven of 50 (14%), a considerable increase in serum hepatocellular enzymes was observed, during chemotherapy, with or without clinical signs of hepatitis. Information about the clinical course, chemotherapeutic regimens and the use of corticosteroids in these seven cases is given in Table 6, while Table 7 summarizes the changes in the serum HbsAg and HbsAb titres together with liver function abnormalities observed during anticancer chemotherapy. Six of seven (86%) patients developed anti-HBs antibodies in their sera with a parallel decrease in the titre of HbsAg (a more detailed presentation concerning these seven patients is planned soon).

In 30 of the 50 (60%) HBsAg carriers, monitoring of HBV profile before, in the middle, just after the completion of chemotherapy and repeatedly during follow-up gave the results shown in Table 8. Two of the 30 HBsAg-positive patients (6.6%) seroconverted to a HBsAg-negative status during their follow-up. Among 28 HBsAb-negative patients, three (11%) developed HBs antibodies. None of the 30 HBcAb-positive patients lost serum

© 1999 Cancer Research Campaign

**Table 6** Clinical course, type of chemotherapy used and use of prednisone in the seven patients who developed HBV reactivation as a result of antineoplastic chemotherapy

| Patient no. | Sex/Age | Diagnosis[a] | Chemotherapeutic regimen (time of HBV reactivation) | Prednisone | Outcome | Resumption of chemotherapy |
|---|---|---|---|---|---|---|
| 1 | M/48 | NHL | CTX+VCR+ADRIA+PRD (3rd course of chemo) | No | Complete recovery | Yes (3 courses) |
| 2 | M/77 | SCLC | CTX+MTX+VCR+VP-16 (6th course of chemo) | Yes | Complete recovery | No |
| 3 | M/48 | SCLC | CTX+MTX+VCR+ADRIA+VP-16 (4th course of chemo) | No | Complete recovery | No |
| 4 | F/63 | NHL | CTX+VCR+ADRIA+PRD (2nd course of chemo) | Yes | Complete recovery | Yes (4 courses) |
| 5 | M/53 | NHL | IFO+VP-16 (4th course of chemo) | No | Complete recovery | Yes (2 courses) |
| 6 | M/67 | APUD | 5-FU+FA (6th course of chemo) | No | Complete recovery | No |
| 7 | M/62 | NHL | CTX+VCR+ADRIA+PRD (3rd course of chemo) | Yes | Complete recovery | Yes (3 courses) |

NHL = Non Hodgkin's lymphoma; SCLC = small-cell lung cancer; APUD = neuroendocrine tumour; CTX = cyclophosphamide; VCR = vincristine; ADRIA = adriamycin; PRD = prednisone; MTX = methotrexate; VP-16 = vepesid IFO = ifosfamide; 5-FU = fluorouracil; FA = folinic acid.

**Table 7** Changes in HBs antigenaemia, titre of serum HBsAb, and liver function tests in the seven HBsAg carriers who developed clinical and/or biochemical evidence of hepatitis during anticancer chemotherapy

| Patient no. | Timing Re: chemo | HbsAg | HBsAb | SGOT | SGPT | γGT | Bilirubin | LDH | PT |
|---|---|---|---|---|---|---|---|---|---|
| 1 | Before | 3500 | –ve | 25 | 25 | 10 | 0.4 | 232 | 11″/11″ |
| | Middle | 2200 | 750 | 340 | 550 | 27 | 0.8 | 491 | 11″/13″ |
| | After | 1500 | 900 | 25 | 28 | 12 | 0.5 | 277 | 11″/11″ |
| 2 | Before | 4000 | –ve | 20 | 20 | 9 | 0.8 | 320 | 11″/11″ |
| | Middle | 4600 | –ve | 25 | 28 | 19 | 0.6 | 270 | 11″/11″ |
| | After | 2600 | 800 | 700 | 500 | 117 | 17 | 1925 | 12″/21″ |
| | Follow-up | –ve | 1300 | 20 | 15 | 23 | 0.4 | 212 | 11″/12″ |
| 3 | Before | 4000 | –ve | 20 | 20 | 10 | 0.65 | 219 | 11″/12″ |
| | Middle | 2800 | 900 | 1900 | 1300 | 56 | 12.5 | 629 | 12″/20″ |
| | After | 2500 | 1000 | 20 | 20 | 19 | 1.25 | 323 | 11″/12″ |
| 4 | Before | 3000 | –ve | 39 | 25 | 12 | 0.7 | 361 | 11″/11″ |
| | Middle | 1600 | 700 | 388 | 315 | 27 | 4.1 | 384 | 11″/16″ |
| | After | 900 | 800 | 28 | 13 | 11 | 0.5 | 170 | 11″/12″ |
| 5 | Before | 2500 | –ve | 20 | 17 | 9 | 0.7 | 150 | 12″/11″ |
| | Middle | 1000 | –ve | 25 | 20 | 10 | 0.8 | 162 | 11″/11″ |
| | After | –ve | 1500 | 140 | 190 | 36 | 2.4 | 468 | 11″/17″ |
| | Follow-up | –ve | 1800 | 18 | 13 | 5 | 1.3 | 170 | 12″/12″ |
| 6 | Before | 1800 | –ve | 26 | 18 | 7.7 | 0.6 | 232 | 13″/12″ |
| | Middle | 1100 | –ve | 22 | 20 | 11 | 0.5 | 228 | 12″/12″ |
| | After | –ve | 1000 | 120 | 115 | 57 | 0.7 | 259 | 13″/11″ |
| | Follow-up | –ve | 1300 | 20 | 15 | 18 | 0.65 | 241 | 12″/11″ |
| 7 | Before | 3100 | –ve | 22 | 17 | 8 | 0.9 | 207 | 12″/11″ |
| | Middle | 3000 | –ve | 24 | 21 | 12 | 0.75 | 205 | 11″/11″ |
| | After | 1600 | –ve | 124 | 129 | 10 | 0.85 | 220 | 11″/12″ |
| | Follow-up | 1500 | –ve | 14 | 16 | 12 | 0.7 | 196 | 11″/11″ |

**Table 8** Monitoring of HBV profile throughout chemotherapy and during follow-up in 30 HBsAg carrier patients

| HBV marker | Before | Middle | After | Follow-up | Overall change (%) |
|---|---|---|---|---|---|
| HBsAg +ve | 30 | 29 | 28 | 28 | –2 (6.6) |
| HBsAb +ve | 2 | 3 | 2 | 5 | +3 (67) |
| HBsAb –ve | 28 | 27 | 28 | 25 | –3 (11) |
| HBcAb +ve | 30 | 29 | 28 | 30 | None |
| HBcAb –ve | 0 | 1 | 2 | 0 | NA |
| HBeAb +ve | 29 | 29 | 29 | 30 | +1 (3.5) |
| HBeAb –ve | 1 | 1 | 1 | 0 | –1 (100) |

 © 1999 Cancer Research Campaign anti-HBc antibodies as a result of anti-neoplastic chemotherapy, while the only HBcAb-negative patient developed serum anti-HBc antibodies during follow-up.

### DISCUSSION

Reactivation of HBV infection leading to clinically evident hepatitis as a result of the administration of antineoplastic chemotherapy, in HBsAg carriers, has been well known from several case reports (Bird et al, 1989; Pinto et al, 1990; Ohtsu et al, 1991; Alexopoulos et al, 1992; Nakamura et al, 1996). Wands et al (1975) surveyed 85 patients with myeloproliferative and lymphoproliferative diseases who were given antineoplastic chemotherapy and observed that, after chemotherapy, HBsAg titres often increased.

Interestingly enough, a systematic evaluation of the incidence of HBV marker positivity, in a large number of untreated patients with solid tumours and a prospective study of the changes of HBV profile, during antineoplastic chemotherapy, has not been published so far. From this point of view, our findings referring to 1008 of 1402 (72%) patients with various solid tumours admitted to the Department of Medical Oncology at Evangelismos Hospital, and to all 920 patients among them who were given antineoplastic chemotherapy, give quite interesting information concerning the prevalence of HBV marker positivity among patients with solid tumours. This information becomes more important considering the fact that the Department of Medical Oncology gets the overwhelming majority of its referrals from the Medical and Surgical Outpatient Departments and the various Inpatient Departments of medical and surgical specialties of Evangelismos Hospital, the biggest general hospital in Greece with an inpatient turnover of 34 000 a year. The findings that 443 out of 1008 patients (44%) had at least one marker of HBV infection positive, provide an estimate of the cumulative incidence of previous exposure to HBV infection of the Greek cancer patients. An identical prevalence (43%) was found among the 920 cancer patients, who received antineoplastic chemotherapy in our Unit.

Even more important, from the point of view of the possibility of developing reactivation of HBV infection, during chemotherapy, was our finding that 50 of these 920 patients (5.4%) were HBsAg carriers. This prevalence is 9 times higher than that of Greek potential blood donors as it has been estimated in a recent multicentre Greek study comprising 542 701 such persons, registered in the Blood Transfusion Units of the five biggest hospitals of Athens in the last 5 years (report in the 1st Hellenic Congress of Sexually Transmittable Diseases). Official data from the Greek Ministry of Health, concerning 70% of the Blood Transfusion Services in Greece, estimate the prevalence of HBsAg carrier state as 0.71%. Nevertheless, this big difference in the prevalence of HbsAg positivity between cancer patients and potential blood donors must be interpreted with caution since blood donors are young and admittedly healthy people most of whom had apparently been self-selected for blood donation knowing their previous HBV profile. Unfortunately there exists no official information about the prevalence of HbsAg carrier state among the general Greek population.

Ohtsu et al (1991), observed a 3.4% HBsAg carrier state among 262 patients with haematologic malignancies, a prevalence not significantly different from the 5.4% observed in our series although patient population in the two studies is not absolutely comparable. Patients in Ohtsu's study had exclusively haematological malignancies and the total number of patients tested was much smaller in his study.

Ngan et al (1995) reported a very high (30%) HBsAg positivity among 73 consecutive Chinese patients with aggressive non-Hodgkin's lymphomas treated between 1988 and 1995, a threefold increase compared with the incidence in general Chinese population. Again this is a very selected population of patients. Of interest are also our findings concerning the evolution of HBV profile after antineoplastic chemotherapy and during follow-up as there were observed in Group C and Group D of patients. While none of the HBs- and HBe-negative patients of Group C developed HBs or HBe antigenaemia during chemotherapy, the overall change for HBsAb marker at the same period was 3.7%, without any evidence of intervening HBV infection or biochemical signs of reactivation of a subclinical hepatitis. The corresponding overall change for HBcAb marker was 4.6%. During the follow-up period (Group D), the biggest changes in the HBV profile were observed in HBeAb and HBcAb markers with overall changes of 16% and 13% respectively.

The evaluation of the behaviour of HBsAg carrier state and the systematic assessment of liver biochemistry, throughout chemotherapy, in the 50 HBsAg-positive patients, together with the findings of monitoring the complete HBV profile before, in the middle, just after completion of chemotherapy and repeatedly during the follow-up in 30 (60%) of them, provide, we believe, valuable information on the possible consequences of antineoplastic chemotherapy and the likelihood of seroconversion in HBsAg carrier patients with solid tumours. An overall 14% of HBsAg carriers (seven patients), developed clinical picture and/or biochemical findings of hepatitis concurrently with the appearance, for the first time, of anti-HBs antibodies in the sera of six of them (persistently in three patients) and an associated decrease in their serum HBsAg levels (persistently in four patients). These findings together with the negative serological results for hepatitis C and D, in all five cases tested, strongly support the explanation that the clinical and/or biochemical hepatitis observed in the seven HbsAg carriers is due to reactivation of HBV infection as a result of cytotoxic chemotherapy.

On the other hand, our finding that among the 30 HBsAg carriers only 6.6% converted to a HBsAg-negative state, persisting during their follow-up, suggests that conversion of HBs antigenaemia as a result of antineoplastic chemotherapy, is neither a frequent nor usually a permanent phenomenon.

Wands et al (1975) reported an increase in serum titre of HBsAg after chemotherapy in all the HBsAg carriers among the 85 patients with haematological malignancies surveyed. Ohtsu et al (1991) observed that five of six HBsAg carriers with haematological malignancies who received chemotherapy containing prednisone, showed a rise in their serum titre of HBsAg. We believe that the difference between their observations and ours is mainly due to the fact that all their patients had haematological malignancies for which high doses of prednisone was given. Furthermore, their findings concern small numbers of patients retroprospectively studied. Our study, involving a significant number of patients followed up prospectively and serially throughout chemotherapy and during follow-up, offers, we think, a more realistic picture of the consequences of administration of antineoplastic chemotherapy in HBsAg carrier patients.

In conclusion, the prevalence of HBsAg carrier state in cancer patients is considerably high in Greece and very significantly higher than that of Greek blood donors. It seems that, during

© 1999 Cancer Research Campaign antineoplastic chemotherapy, one out of seven carriers will develop reactivation of HBV infection. It would, therefore, be advisable to screen all cancer patients for HbsAg prior to chemotherapy. Although the findings would probably not affect the decision about management, it seems prudent to closely follow those patients screened positive for the possibility of developing severe hepatitis.

Finally, disappearance of HBs antigenaemia following chemotherapy induced reactivation of HBV infection is an infrequent and usually transient phenomenon.

### REFERENCES

Alexopoulos CG, Vaslamatzis M, Zoublios C and Chatzidimitriou G (1992) Reactivation of hepatitis B in HBsAg carriers during cancer chemotherapy. *Ann Oncol* **3**: 65

Alexopoulos CG, Vaslamatzis M and Chatzidimitriou G (1997) Incidence of hepatitis B virus (HBV) positivity and behaviour of hepatitis B surface antigen (HBsAg) carrier state in cancer patients, during chemotherapy (CT). *Proc Am Soc Clin Oncol* **16**: A1490

Bird GLA, Smith H, Portmann B, Alexander GJM and Williams R (1989) Acute liver decompensation on withdrawal of cytotoxic chemotherapy and immunosuppressive therapy in hepatitis B carriers. *Quart J Med, New Series 73* **270**: 895–902

Gallbraith RM, Eddleston AL, Williams R, Zucherman AJ and Bagshaw KD (1975) Fulminant hepatic failure in leukemia and choriocarcinoma related to withdrawal of cytotoxic drug therapy. *Lancet* **2**: 528–530

Hoofnagle JH, Dusheiko GM, Schafer DF, Jones EA, Micetich CK, Young CR and Costa J (1982) Reactivation of chronic hepatitis B virus infection by cancer chemotherapy. *Am Intern Med* **96**: 447–449

Lau JYN, Lai CL, Lin HJ, Lok ASF, Liang RHS, Wu CP, Chan KT and Todd D (1989) Fatal reactivation of chronic hepatitis B virus infection following withdrawal of chemotherapy in lymphoma patients. *Quart J Med New Series 73* **270**: 911–917

Nakamura Y, Motokura T, Fujita A, Yamashita T and Ogata E (1996) Severe hepatitis related to chemotherapy in hepatitis B virus carriers with hematologic malignancies. Survey in Japan 1987–1991. *Cancer* **78**: 2210–2215

Ngan R, Tung S, Lau WH and Lam J (1995) Long term results of aggressive lymphoma using PROMACE-CYTABOM, peculiar patient group and toxicity. *Proc Am Soc Clin Oncol* **14**: 1251

Ohtsu T, Sai T, Oka M, Shgai Y and Tobinai K (1991) Activation of hepatitis B virus infection by chemotherapy containing glucocorticoid in hepatitis B virus carriers with hematologic malignancies. *Jpn J Clin Oncol* **21**: 360–365

Pinto CP, Hu E, Bernstein-Singer M, Pinter-Brown L and Govindarajans (1990) Acute hepatic injury after withdrawal of immunosuppressive chemotherapy in patients with hepatitis B. *Cancer* **65**: 878–884

Scullard GH, Smith CI, Merigan TC, Robinson WS and Gregory PB (1981) Effects of immunosuppressive therapy on viral markers in chronic active hepatitis B. *Gastroenterology* **81**: 987–991

Wands JR, Chura CM, Roll FJ and Maddrey WC (1975) Serial studies of hepatitis-associated antigen and antibody in patients receiving antitumor chemotherapy for myeloproliferative and lymphoproliferative disorders. *Gastroenterology* **68**: 105–112

*© 1999 Cancer Research Campaign*
